# OFDR Distributed Demodulation Optimization Algorithm Using Discrete-Time Analytic Signal Backscattered Rayleigh Spectrum

**DOI:** 10.3390/s25227044

**Published:** 2025-11-18

**Authors:** Shuaipeng Wang, Haomao Wang, Zhiguo Zhang, Yifan Wang, Haichao Huang

**Affiliations:** 1 Beijing Smart-Chip Microelectronics Technology Co., Ltd., Beijing 100192, China; wangshuaipeng@sgchip.sgcc.com.cn (S.W.); huanghaichao@sgchip.sgcc.com.cn (H.H.); 2 State Key Laboratory of Information Photonics and Optical Communications, Beijing University of Posts and Telecommunications, Beijing 100876, China; wanghaomao@bupt.edu.cn (H.W.); wangyifan7693@bupt.edu.cn (Y.W.)

**Keywords:** OFDR, Rayleigh, backscattering spectrum, discrete time analytic signal

## Abstract

We propose a novel distributed demodulation optimization algorithm for optical frequency domain reflectometry (OFDR). This algorithm applies discrete-time analytic (DTA) signals to the Rayleigh backscattered signal (RBS) reconstruction. The DTA-RBS algorithm utilizes only positive-frequency components in the distance domain and employs a frequency-domain construction method to generate DTA-RBS, thereby improving performance without increasing the computational complexity of the OFDR demodulation algorithm. By leveraging the envelope property, DTA-RBS enhances spectral feature information and intensity while effectively suppressing high-frequency noise and spurious oscillations introduced during reconstruction, thereby maintaining a higher correlation between the reference and test data. Comprehensive experimental validation demonstrates significant performance improvements across multiple metrics. Cross-correlation intensity analysis shows that the average peak intensity of DTA-RBS reaches 0.9527, compared to 0.9096 for the conventional method. Standard deviation measurements on unstrained fiber segments demonstrate a 63% improvement. Large-strain demodulation experiments show that DTA-RBS exhibits superior strain demodulation performance and robustness, whereas the conventional method produces anomalous data points due to false peaks obscuring genuine correlation peaks. These results confirm that the DTA-RBS method provides a theoretically rigorous and practically effective approach for enhancing the sensing accuracy, stability, and robustness of OFDR in high-precision distributed measurement applications.

## 1. Introduction

Optical Frequency Domain Reflectometry (OFDR) has emerged as a powerful distributed sensing technology distinguished by its high spatial resolution [1] and measurement precision [2], finding extensive applications across diverse industrial domains. In aerospace engineering, OFDR enables deformation monitoring of array antenna systems [3], providing critical feedback for maintaining structural integrity and optimal performance. The technology has proven equally valuable in power infrastructure, where it facilitates real-time temperature monitoring of high-voltage switchgear [4], enhancing operational safety and preventing potential failures. In the field of energy infrastructure monitoring, OFDR is employed for temperature monitoring of insulation oil-immersed commercial distribution transformers [5], ensuring reliable operation of critical power distribution equipment. These practical applications impose stringent requirements on OFDR systems regarding sensing accuracy, measurement range, demodulation speed, and long-term stability, driving continuous advancement in demodulation algorithms and signal processing techniques.

The fundamental demodulation process of OFDR comprises three key stages [6]. Distance domain mapping is achieved through Fast Fourier Transform (FFT), followed by sliding window [7] selection of distance domain data and reconstruction of the Rayleigh backscattering spectrum (RBS) [8], and finally wavelength shift corresponding to strain or temperature changes is extracted through cross-correlation calculation [9]. Among these stages, the quality of the reconstructed RBS serves as a critical determinant of overall demodulation performance. The RBS reconstruction process involves extracting distance domain data through a sliding window, applying zero-padding operations to enhance spectral resolution, and performing the Inverse Fast Fourier Transform to obtain local spectral information. However, this conventional reconstruction workflow inherently introduces multiple sources of error and degradation. The sliding window truncation operation inevitably produces spectral leakage and edge effects in the frequency domain [10]. Zero-padding operations, while improving spectral resolution, may introduce ringing artifacts [11] in the reconstructed spectrum. Furthermore, the finite-length nature of the data reconstruction process generates truncation errors [12] that manifest as high-frequency oscillations and spurious peak fluctuations in the local spectrum. These combined effects compromise the quality of the reconstructed RBS, introducing high-frequency noise [13] components that adversely affect subsequent cross-correlation calculations and ultimately degrade the accuracy and stability of distributed sensing measurements.

Current research efforts have focused on optimizing RBS reconstruction to enhance OFDR sensing performance using various approaches. In 2018, Cui et al. investigated methods involving zero-padding and interpolation techniques applied during the RBS reconstruction process to improve sensing accuracy [14]. While these approaches demonstrated measurable improvements, the enhancement in sensing precision remained limited despite the substantial increase in computational complexity associated with extended zero-padding operations and sophisticated interpolation algorithms. More recently in 2024, Wang et al. applied FPGA-based acceleration to OFDR demodulation data processing, implementing high-precision real-time OFDR sensing through rational configuration of RBS zero-padding points and interpolation algorithms [15]. However, the contribution of this study to RBS quality improvement remains limited to parameter optimization for efficient demodulation, without introducing innovative enhancements to the RBS itself. Additionally, in 2024, Yu et al. proposed an alternative methodology utilizing dual-segment RBS similarity features [16]. This approach computes cross-correlation results between two RBS segments and employs appropriate threshold criteria to optimize strain range and measurement repeatability. Nevertheless, this method does not fundamentally improve the intrinsic quality of RBS but rather trades increased computational burden for enhanced reliability through redundant measurement and validation mechanisms. These existing approaches highlight the persistent challenge of achieving significant performance improvements without proportionally increasing algorithm complexity or computational requirements.

The present study addresses this fundamental challenge by proposing an innovative methodology that improves RBS quality while maintaining computational efficiency. We introduce a reconstruction method based on discrete-time analytic (DTA) signals applied to the RBS reconstruction process. The resulting DTA-RBS is employed for OFDR distributed demodulation. The DTA signal approach leverages the fundamental mathematical property that the magnitude of an analytic signal corresponds to the envelope of the original real signal. This envelope extraction mechanism offers inherent advantages for suppressing reconstruction artifacts and noise. During the OFDR demodulation process, the distance domain data selected by the sliding window can be directly transformed into the frequency domain representation of the DTA signal through simple scaling operations, requiring only positive frequency domain components without necessitating conjugate symmetric data. This streamlined approach does not increase computational complexity compared to conventional methods. The resulting DTA-RBS exhibits envelope characteristics that highlight the characteristic information of the spectrum while effectively filtering high-frequency noise and spurious oscillations introduced during truncation, zero-padding, and finite-length reconstruction processes. This dual benefit of feature enhancement and error suppression yields higher-quality local spectral data for cross-correlation calculations. Furthermore, the elimination of conjugate symmetric data requirements reduces the addressing range by half in FPGA-based implementations, offering significant advantages for hardware resource utilization and system optimization. Through comprehensive experimental validation, we demonstrate that the DTA-RBS method achieves substantial improvements in cross-correlation intensity, standard deviation performance, and strain demodulation reliability, providing a theoretically sound and practically effective optimization pathway for enhancing OFDR sensing capabilities in high-precision distributed measurement applications.

## 2. Principles and Methods

### 2.1. Principle of OFDR

**Figure 1:**
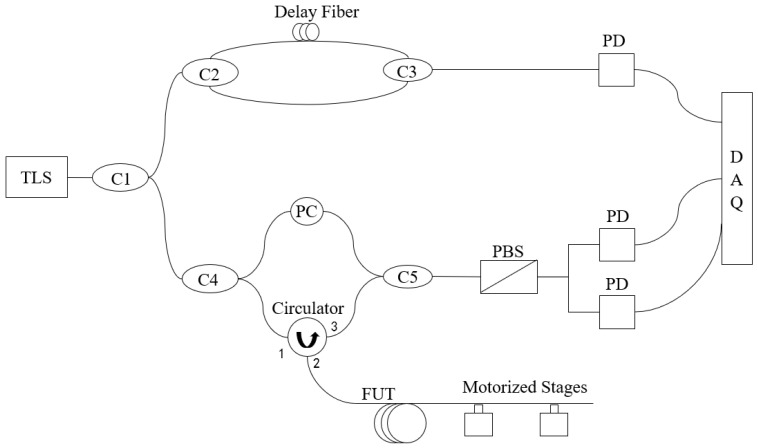
OFDR System Schematic.TLS is tunable laser source. PD is balanced photodetector. DAQ is data acquisition card. PC is polarization controller. PBS is polarization beam splitter. FUT is fiber under test. C1-C5 are couplers. Ports 1, 2, and 3 are the port numbers of the circulator, where the TLS signal enters at port 1 and exits at port 2, and the Rayleigh backscattered signal from the FUT enters at port 2 and exits at port 3.

OFDR is a distributed sensing technology based on fiber Rayleigh backscattering [17]. In this study, the structure of the OFDR system is shown in Figure 1. A Luna Phoenix 1200 was selected as the tunable laser source (TLS). The optical path consists of couplers with different coupling ratios (C1, C4 1:99; C2, C3, C5 50:50), circulators, polarization controllers (PC), and polarization beam splitters (PBS). A balanced photodetector (PD) was used for optical-to-electrical signal conversion, with signal acquisition performed on a data acquisition card (DAQ). This research employed a hardware compensation method [18] to compensate for the nonlinear effects of laser frequency sweeping [19]. The fiber under test (FUT) was 50 m in length, while a delay fiber was used to create an optical path difference of 200 m in the auxiliary interferometer.

Assume a reflection point of the fiber under test (FUT) at a distance $z_{i}$ from the circulator.$\tau_{i}$ is the time delay of the Rayleigh backscatter signal (RBS) at position $z_{i}$, which can be expressed as follows:

(1) $$\tau_{i}=\frac{2nz_{i}}{c},$$

where *c* is the speed of light in vacuum, and *n* is the refractive index of the fiber. The frequency tuning rate of the TLS is γ. The beat signal generated by the main interferometer is detected at the BPD. The beat current detected by the BPD can be expressed as follows:

(2) $$I_{b}\left(t\right)=\sum_{i=0}^{N}2\sqrt{R\left(\tau_{i}\right)}E_{0}^{2}\cos\left[2\pi \left(f_{0}\tau_{i}+\gamma \tau_{i}t-\frac{1}{2}\gamma \tau_{i}^{2}\right)+\varphi \left(\tau_{i}\right)\right],$$

where $f_{0}$ is the initial frequency of the TLS, and $\varphi \left(\tau_{i}\right)$ is the phase variation caused by strain or temperature along the FUT. $E_{0}$ is the amplitude of the reference signal. $R\left(\tau_{i}\right)$ is the Rayleigh scattering coefficient.

As shown in Equation (2), after performing FFT on the sampled data, the frequency domain information obtained carries the distance information of the test fiber. Therefore, frequency-domain data is also referred to as distance-domain data. Subsequently, to implement distributed sensing, a sliding window of length N is utilized to extract the same distance domain data segment from both the reference data and test data. It should be noted that in the conventional demodulation process, since the collected signal is a real signal, the Fourier transform possesses conjugate symmetry. Therefore, when implementing the sliding window, it is necessary to select conjugate-symmetric distance-domain data for subsequent reconstruction. The length of the sliding window N determines the spatial resolution of the distributed demodulation results. Zero-padding is applied to the sliding window data to increase the wavelength resolution. Through the Inverse Fast Fourier Transform (IFFT), the RBS of the corresponding test fiber segment is obtained. Cross-correlation operations are performed on the RBS of the reference and test data to determine the offset of the maximum peak of the cross-correlation function, which corresponds to the wavelength shift. The wavelength shift is proportional to changes in strain or temperature. By iteratively traversing the sliding window throughout the entire test fiber and calculating the RBS and wavelength shift, the distributed demodulation results of the test fiber can be obtained.

### 2.2. Principle of Discrete-Time Analytic Signal

A discrete-time analytic (DTA) signal is a complex-valued sequence derived from a real-valued signal, possessing the fundamental property that its discrete-time Fourier transform (DTFT) vanishes for negative frequencies [20]. This one-sided spectral characteristic eliminates redundant conjugate-symmetric components, making DTA signals particularly valuable for applications requiring bandwidth compression and complex signal representation.

For a finite-length real-valued sequence x(n) of even length N, the corresponding DTA signal z(n) is formally defined through the Hilbert transform as shown in Equation (3):

(3) $$\begin{array}{c} z(n)=x(n)+jH\{x(n)\}. \end{array}$$

where H denotes the Hilbert transform operator and $j=\sqrt{-1}$. The analytic property is mathematically expressed in the frequency domain by Equation (4):

(4) $$Z(e^{j\omega })=0\;\mathrm{for}\;-\pi \leq \omega <0.$$

Due to the continuous nature of the DTFT, practical implementation requires the DTF. The standard frequency-domain approach for generating DTA signals follows a systematic three-step procedure. First, the N-point DFT of the real signal is computed as per Equation (5):

(5) $$X\left(k\right)=\sum_{n=0}^{N-1}x\left(n\right)e^{-j\frac{2\pi }{N}kn},\;k=0,1,\ldots ,N-1.$$

The DFT of the DTA signal is then constructed through spectral modification according to Equation (6):

(6) $$Z\left(k\right)=\left\{\begin{array}{cc} X(0) & \mathrm{for}\;k=0\\ 2X(k) & \mathrm{for}\;k=1,2,\ldots ,\frac{N}{2}-1\\ X\left(\frac{N}{2}\right) & \mathrm{for}\;k=\frac{N}{2}\\ 0 & \mathrm{for}\;k=\frac{N}{2}+1,\ldots ,N-1. \end{array}\right.$$

Finally, the DTA signal is recovered through the inverse DFT operation defined in Equation (7):

(7) $$z\left(n\right)=\frac{1}{N}\sum_{k=0}^{N-1}Z\left(k\right)e^{j\frac{2\pi }{N}kn},\;n=0,1,\ldots ,N-1.$$

The time-domain expression for z(n) can be explicitly derived as having different forms for even and odd indices. For even indices n, the expression is given by Equation (8):

(8) $$z\left(n\right)=x\left(n\right)+j\left[\frac{2}{N}\sum_{m=0}^{N-1}x\left(m\right)\cot\left(\frac{\pi }{N}\left(n-m\right)\right)\right]\;\mathrm{for}\;n\;\mathrm{even}.$$

For odd indices n, the expression is given by Equation (9):

(9) $$z\left(n\right)=x\left(n\right)+j\left[\frac{2}{N}\sum_{m=0}^{N-1}x\left(m\right)\cot\left(\frac{\pi }{N}\left(n-m\right)\right)\right]\;\mathrm{for}\;n\;\mathrm{odd}.$$

Both expressions contain the cotangent summation term $\frac{2}{N}\sum_{m=0}^{N-1}x\left(m\right)\cot\left(\frac{\pi }{N}\left(n-m\right)\right)$, which represents the discrete Hilbert transform component.

This conventional DFT-based method exhibits a critical theoretical limitation where it fails to generate a genuine complex analytic signal for a specific class of signals where all even-indexed values equal some real constant and all odd-indexed values equal a different real constant, both constants being nonzero. For such signals, the imaginary component becomes identically zero, causing the analytic mapping to degenerate to an identity transformation, resulting in purely real output z(n)=x(n) rather than complex analytic representation. This failure occurs because signals of this specific type have DFTs containing at most frequency components at DC (k = 0) and Nyquist (k = N/2) frequencies, leading to complete cancellation of the imaginary part [21]. However, in OFDR demodulation processes, where sliding windows reconstruct local spectra, this particular special case that causes method failure does not occur, making the frequency-domain method directly applicable for establishing discrete-time analytic signals in OFDR demodulation.

**Figure 2:**
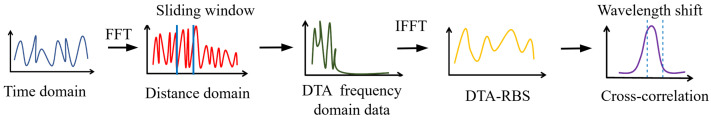
OFDR demodulation process and the construction flowchart of DTA-RBS.

The OFDR demodulation process and the construction flowchart of DTA-RBS are shown in Figure 2. Time-domain data is mapped to the distance domain through FFT. According to the DTA signal principle, distance-domain data is selected using a sliding window. After coefficient scaling of the distance-domain data according to Equation (6), DTA frequency-domain data is constructed. Subsequently, IFFT is performed on the frequency-domain data and the magnitude is extracted to obtain the DTA-RBS. Finally, through cross-correlation, the wavelength shift between the reference and test data is calculated, and the distributed demodulation result is obtained.

A fundamental property of analytic signals is the equivalence between the magnitude of the analytic signal and the envelope of the original real signal. For discrete-time signals, this relationship is mathematically established through the magnitude calculation shown in Equation (10):

(10) $$\left|z\left(n\right)\right|=\sqrt{x^{2}\left(n\right)+{\left[H\left\{x\left(n\right)\right\}\right]}^{2}}.$$

This relationship holds under the condition that x(n) is a bandlimited signal with appropriate spectral characteristics. The discrete Hilbert transform H{x(n)} effectively creates a quadrature component that, when combined with the original signal through the magnitude operation, yields the instantaneous amplitude. The squared magnitude operation in time domain corresponds to convolution in frequency domain as expressed in Equation (11):

(11) $${\left|z\left(n\right)\right|}^{2}=z\left(n\right)z^{*}\left(n\right)⟺Z\left(\omega \right)*Z^{*}\left(-\omega \right).$$

Due to the one-sided nature of Z(ω), this operation effectively extracts the low-frequency components that constitute the signal envelope. For narrowband signals where the envelope varies slowly compared to the carrier frequency, the magnitude |z(n)| provides an excellent approximation to the true signal envelope.

This mathematical relationship has substantial practical significance across various applications. In the context of OFDR demodulation, the use of DTA signals provides particularly valuable benefits. The analytic signal representation reduces signal distortion and high-frequency noise [22] introduced during the IFFT process, which is crucial for maintaining signal integrity in precision measurement applications. Additionally, DTA signals enhance the characteristic information and intensity of local spectra, improving the resolution and accuracy of distributed sensing measurements. The mathematical rigor ensures the analytic signal approach provides a theoretically sound foundation for envelope extraction with well-defined validity and accuracy conditions, making it particularly suitable for high-precision optical frequency domain reflectometry applications where accurate signal envelope detection is essential for spatial resolution and measurement accuracy.

In the OFDR demodulation process, the advantage of the DTA-RBS method over the RBS method lies in the fact that DTA-RBS strengthens spectral features and intensity without increasing algorithm complexity. During OFDR demodulation, the frequency-domain conversion of data has already been completed, and the positive frequency-domain data can be directly selected using a sliding window for scaling processing to construct the DTA-RBS frequency-domain data. The construction of RBS requires this step as well, and necessitates conjugate symmetric frequency-domain data. Therefore, in the sliding window step, DTA-RBS and RBS share the same procedure, and no additional computational complexity is introduced to the demodulation process. In the spectral processing phase, conventional algorithms perform a series of operations on the RBS, including peak detection, interpolation, and smoothing, to ensure spectral quality. However, DTA-RBS possesses an envelope property that enhances spectral features, eliminating the need for additional processing steps before subsequent demodulation. Therefore, DTA-RBS demonstrates an advantage in algorithm complexity compared to RBS.

This characteristic manifests even greater advantages in FPGA-based OFDR systems. Since constructing DTA-RBS requires only positive frequency-domain data, it optimizes storage space by 50%, which substantially reduces the hardware memory burden on the FPGA. During the sliding window selection of frequency-domain data, RBS requires conjugate symmetric frequency-domain data, necessitating complex addressing logic in the FPGA data reading process. In contrast, DTA-RBS only requires sequential reading of positive frequency-domain data, simplifying the addressing logic and optimizing 50% of the addressing range, which is significant for FPGA logical design and layout routing optimization. Furthermore, the envelope property of DTA-RBS enhances spectral feature information and intensity, allowing the FPGA processing to eliminate steps such as linear interpolation and smoothing filtering. The optimization of these computationally intensive algorithms reduces the consumption of logical resources and is important for overall demodulation speed.

## 3. Experiments and Results Analysis

In the experiments of this study, the test fiber had a length of 50 m and was selected as a single-mode fiber. An auxiliary interferometer with an optical path difference of 200 m was employed to compensate for the nonlinear effects of source frequency sweep. The tunable laser source (TLS) was selected as LUNA1200, which features a tuning range of 1515 nm to 1565 nm, a maximum sweep rate of 1000 nm/s, and a spectral linewidth of 1.5 MHz. In the subsequent experiments, the tuning range of the TLS was set to 1546–1554 nm, spanning a total of 8 nm. The sweep rate was set to 100 nm/s. The photodetector was selected as Thorlabs PDB465C-AC with a bandwidth of 150 MHz. The data acquisition card was a GAGE CSE8329 with dual-channel 14-bit resolution and a maximum sampling rate of 150 MS/s. The number of sampling points was set to 2 M. At 47 m from the end of the test fiber, the sample was rigidly fixed on a translation stage for subsequent distributed strain measurements. The strain loading region of the motorized stage had a length of 80 cm. During the OFDR distributed demodulation process, the length of the distance domain sliding window was set to 64 points, corresponding to a spatial resolution of 3.2 mm. During the reconstruction of the RBS and DTA-RBS, the spectral length was set to 2048 points.

### 3.1. Reconstruction of RBS and DTA-RBS

To investigate the properties and effectiveness of DTA-RBS, this study selected data in the distance domain at 3.2 m of the test fiber for a comparative analysis of the reconstruction processes of RBS and DTA-RBS. In the data processing workflow, the distance-domain data is first extracted through a sliding window, followed by zero-padding operations to synthesize frequency-domain data.

**Figure 3:**
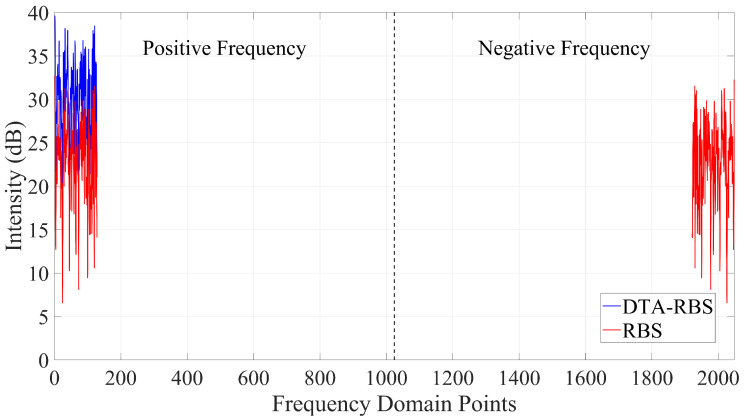
Frequency domain comparison of RBS and DTA-RBS. The dashed line appears at the center of the frequency domain data, representing the boundary between the positive and negative frequency domains.

Figure 3 shows the differences in the frequency domain data between RBS and DTA-RBS. The frequency domain data of RBS exhibit conjugate symmetry characteristics, with data distributed in both positive and negative frequency domains, which is a characteristic of frequency domain data obtained from real signals after Fourier transform. In contrast, DTA-RBS retains valid data only in the positive frequency domain, with the negative frequency components set to zero, and the amplitude of the positive frequency data is twice that of RBS, achieving a single-sided spectrum representation.

**Figure 4:**
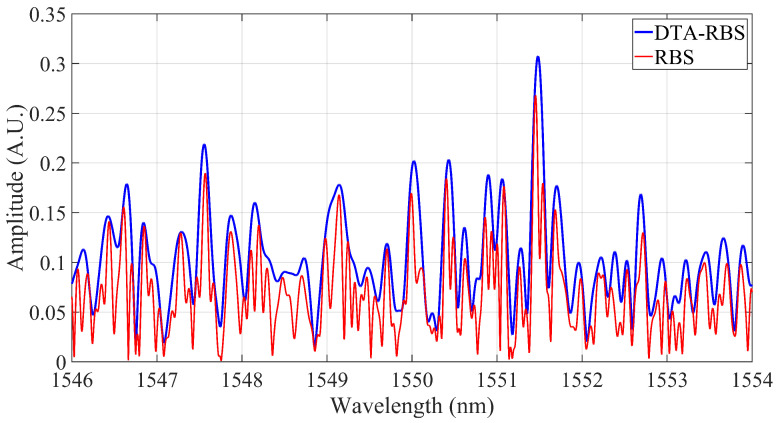
Comparison of RBS and DTA-RBS reconstruction results.

After inverse fast Fourier transform (IFFT) processing, the RBS and DTA-RBS are shown in Figure 4. DTA-RBS is morphologically equivalent to the overall envelope of RBS. A single peak in DTA-RBS often contains information from multiple adjacent peaks in RBS and exhibits greater peak intensity. There is a clear correspondence between the two spectral representations. DTA-RBS highlights the overall characteristics of the spectrum through its envelope properties and enhances the overall intensity of the spectrum.

From a signal processing perspective, in the processing workflow of frequency-domain data truncation, zero-padding, and inverse fast Fourier transform, the traditional RBS reconstruction method is prone to introduce multiple types of errors. The truncation operation of frequency-domain data by the sliding window inevitably produces spectral leakage and edge effects. Zero-padding operations may introduce ringing effects after inverse transformation. Moreover, the reconstruction process of finite-length data generates truncation errors, leading to high-frequency oscillations and spurious peak fluctuations in the local spectrum, causing the reconstructed local spectrum to exhibit high-frequency noise characteristics. The envelope property exhibited by DTA-RBS is similar to applying an adaptive smoothing process to the signal, which can effectively filter out high-frequency noise and spurious oscillations introduced by truncation, zero-padding, and finite-length reconstruction. This dual effect of feature enhancement and error suppression provides higher-quality local spectral data for subsequent cross-correlation calculations and distributed sensing demodulation. We will analyze its performance advantages in detail in the following sections.

### 3.2. Cross-Correlation Intensity Experiments of RBS and DTA-RBS

To investigate the improvement effect of DTA-RBS on cross-correlation intensity, this study selected data at 10.24 m of the test fiber for detailed analysis. No strain was applied at this position, and theoretically, the cross-correlation results between the reference data and test data should not produce wavelength shift.

**Figure 5:**
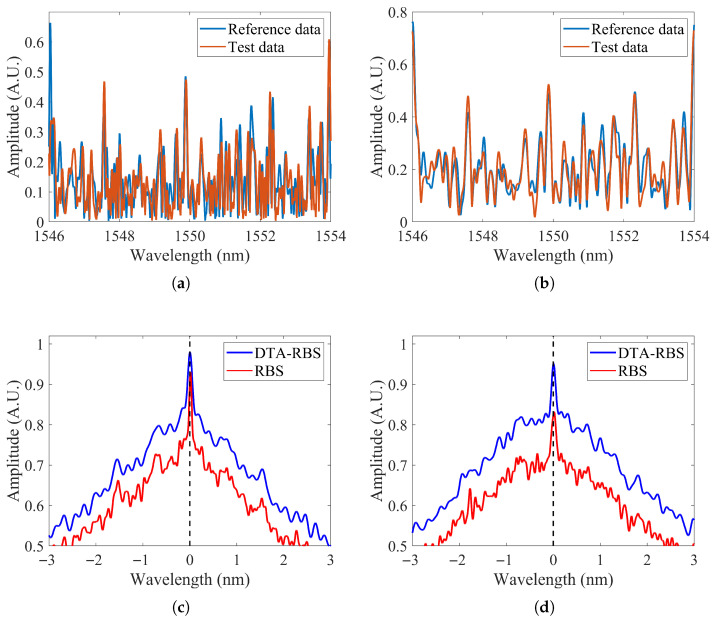
(**a**) RBS of reference and test data at 10.24 m. (**b**) DTA-RBS of reference and test data at 10.24 m. (**c**) Cross-correlation results of RBS and DTA-RBS at 10.24 m. (**d**) Cross-correlation results of RBS and DTA-RBS at 48 m.

Figure 5a shows the RBS reconstruction results of the reference data and the test data at 10.24 m from the test fiber. Figure 5b shows the DTA-RBS reconstruction results of the reference data and test data at the same position. Comparing the two figures reveals distinct differences. The RBS in Figure 5a exhibits a higher peak density and contains high-frequency oscillation components. Careful observation shows that some peak positions in the RBS spectra of the reference data and test data overlap, but a considerable number of peak positions fail to achieve effective matching. This matching inconsistency originates from high-frequency noise and spurious peak fluctuations introduced during the reconstruction process. In contrast, the DTA-RBS in Figure 5b demonstrates superior peak position matching characteristics, with significantly improved waveform similarity between the reference data and the test data. This improvement directly reflects the effective extraction capability of DTA-RBS for spectral features through its envelope properties.

Figure 5c shows the comparison results of cross-correlation functions between RBS and DTA-RBS at 10.24 m of the test fiber. From the overall distribution, the cross-correlation function intensity of DTA-RBS is significantly higher than that of RBS. Quantitative analysis shows that the cross-correlation peak intensity of DTA-RBS reaches 0.977, while the cross-correlation peak intensity of RBS is only 0.936. The improvement in cross-correlation intensity of DTA-RBS can be explained from multiple perspectives. First, DTA-RBS effectively suppresses high-frequency noise and spurious oscillations introduced during the reconstruction process through envelope extraction, making the spectral features of the reference data and test data more stable and possessing higher intensity. Second, by filtering out distortion components caused by truncation, zero-padding, and finite-length inverse transformation, DTA-RBS highlights the main feature information of the local spectrum and enhances the similarity between the reference spectrum and test spectrum. This feature enhancement effect enables DTA-RBS to more accurately identify and match spectral features in cross-correlation calculations, thereby obtaining a higher cross-correlation peak intensity.

To further verify the performance of DTA-RBS under degraded signal-to-noise ratio conditions, Figure 5d shows the cross-correlation intensity comparison results at 48 m of the distal end of the test fiber. At the distal end of the test fiber, the signal-to-noise ratio decreases due to transmission loss and natural attenuation of Rayleigh backscattering signals. Under these unfavorable conditions, the cross-correlation peak intensity of DTA-RBS still maintains a relatively high level of 0.951, while the cross-correlation peak intensity of RBS decreases to 0.843. This comparison result fully demonstrates that the DTA-RBS method can still maintain good cross-correlation performance in low signal-to-noise ratio environments, exhibiting stronger noise resistance and more stable feature extraction effects.

**Figure 6:**
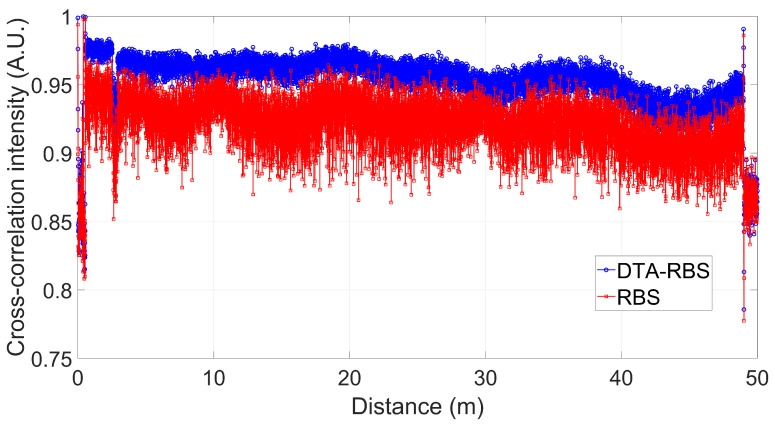
Cross-correlation peak intensity distribution of RBS and DTA-RBS along the test fiber.

Figure 6 shows the comparison of the intensity distributions of the cross-correlation between RBS and DTA-RBS during the distributed demodulation process of the entire test fiber. From the overall trend, the cross-correlation intensity of DTA-RBS consistently maintains a high level throughout the entire length of the test fiber and is overall superior to RBS. Statistical analysis shows that the average cross-correlation peak intensity of DTA-RBS is 0.9527, while the average cross-correlation peak intensity of RBS is 0.9096. This systematic performance improvement demonstrates that the DTA-RBS method has universal applicability and stability in the entire fiber optic sensing system. Higher cross-correlation intensity means stronger correlation between the reference data and test data, which will directly translate into more accurate wavelength shift calculations and more reliable strain demodulation results, establishing a solid foundation for improving the overall measurement accuracy of OFDR.

### 3.3. STD Performance Experiments of RBS and DTA-RBS

To evaluate the improvement effect of DTA-RBS on distributed sensing stability, this study selected the distributed sensing demodulation results in the range of 30 m to 43 m of the test fiber for in-depth analysis. No strain was applied to this fiber section, and theoretically, the demodulation results should not produce wavelength shift. However, in actual measurement processes, influenced by system noise and environmental disturbances, the demodulation results exhibit random fluctuations to a certain degree. By quantifying these fluctuation characteristics through standard deviation, the stability and reliability of the sensing system can be effectively evaluated.

**Figure 7:**
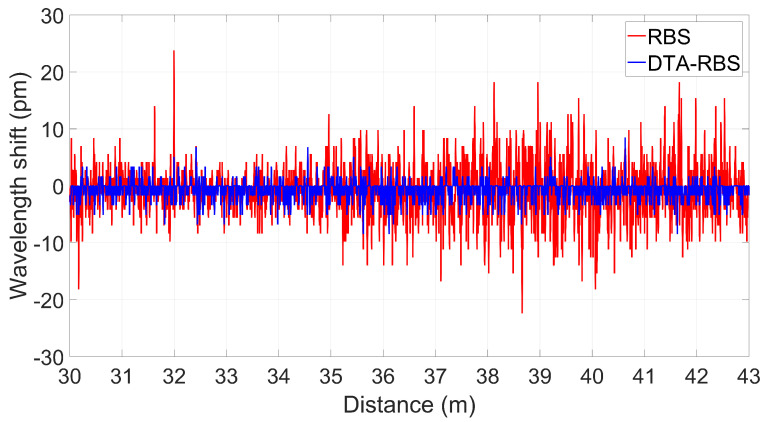
Distributed sensing results from 30 m to 43 m of the test fiber.

Figure 7 shows the comparison of distributed sensing demodulation results between RBS and DTA-RBS in the range of 30 m to 43 m of the test fiber. From overall trend observation, the fluctuation range of DTA-RBS demodulation results is significantly smaller than that of RBS demodulation results. Along the fiber transmission direction, as the demodulation distance increases, the Rayleigh backscattering signal experiences a longer transmission path, and the accumulated transmission loss leads to a gradual decrease in signal-to-noise ratio. Under these conditions, the fluctuation amplitude of RBS demodulation results shows a gradually increasing trend, reflecting its sensitivity to signal-to-noise ratio degradation. However, it is noteworthy that the demodulation fluctuations of DTA-RBS are almost unaffected by the decrease in signal-to-noise ratio and consistently maintain a small fluctuation range. This phenomenon fully demonstrates the demodulation stability advantage of the DTA-RBS method throughout the entire measurement range. In the range of 30 m to 43 m of the test fiber, the STD of RBS demodulation results is 7.7808 pm, while the STD of DTA-RBS demodulation results is only 2.8267 pm, achieving a 63% improvement in STD performance.

DTA-RBS highlights the main feature information of the local spectrum while filtering out local high-frequency detail fluctuations. This feature enhancement mechanism makes the correlation between the reference data and test data more stable, thereby reducing random errors in wavelength shift calculations. More importantly, the higher cross-correlation peak intensity of DTA-RBS provides more accurate correlation peak position identification, reduces the uncertainty of peak localization, and thereby improves the stability of the entire demodulation process. These factors work together to enable DTA-RBS to maintain good noise resistance and stable demodulation performance when facing unfavorable conditions such as signal-to-noise ratio degradation.

**Figure 8:**
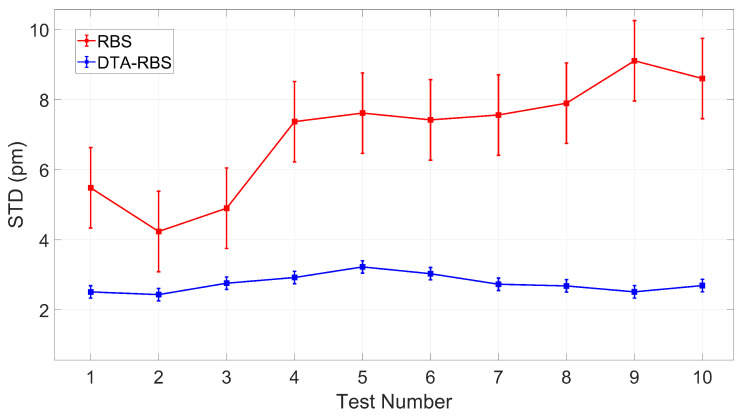
STD of distributed sensing results over 10 measurements of the test fiber.

To further verify the stability and repeatability of the DTA-RBS method, this study conducted 10 independent tests on the distributed optical fiber and systematically analyzed the STD performance of the demodulation results. As shown in Figure 8, quantitative comparison with error bars and confidence intervals reveals significant performance differences between the two methods.

The RBS method yielded an average STD of 7.0196 ± 1.1476 pm (95% confidence interval), while the DTA-RBS method achieved a substantially lower average STD of 2.7523 ± 0.1776 pm (95% confidence interval), representing an improvement of 4.2673 pm (60.79%). A paired *t*-test was performed to evaluate the statistical significance of this performance difference, yielding t(9) = 8.70, *p* < 0.001. This result demonstrates that the performance difference between the two algorithms is statistically significant at the *p* < 0.05 level.

The wider confidence interval of RBS (±1.1476 pm) indicates substantial performance fluctuation across different measurements, reflecting its sensitivity to variations in measurement conditions. In contrast, the narrow confidence interval of DTA-RBS (±0.1776 pm) demonstrates consistently low and stable STD performance throughout all 10 tests with minimal inter-test variability. This superior consistency and stability evidence that the DTA-RBS method exhibits excellent robustness and provides reliable demodulation results across varying measurement conditions, thereby ensuring dependable long-term monitoring performance for practical field applications.

### 3.4. Distributed Strain Demodulation Experiments of RBS and DTA-RBS

**Figure 9:**
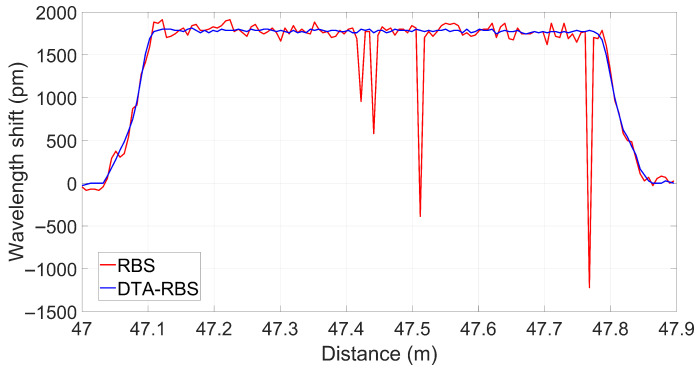
Distributed sensing results of the strain region demodulated by RBS and DTA-RBS.

To verify the demodulation performance of DTA-RBS in practical strain measurement, a 80 cm section of optical fiber from 47 m to 47.8 m at the fiber end was securely attached to Thorlabs motorized stages. Reference data were collected in a stable state without strain. Subsequently, the motorized stages were controlled to stretch the optical fiber by 1.2 mm, corresponding to applying 1500 μϵ to the test fiber. Test data were collected in the stable state. The strain region was demodulated separately using RBS and DTA-RBS methods, and the demodulation results are shown in Figure 9.

Significant performance differences can be observed from the overall demodulation results. Under large strain conditions, the distributed demodulation results of RBS exhibited four anomalous data points in the strain region. The demodulation values of these anomalous points significantly deviated from the true level of applied strain, indicating that demodulation failure occurred at these positions. In contrast, DTA-RBS achieved correct demodulation at all positions throughout the entire strain region without any anomalous data points. Additionally, within the strain region, the demodulation results of RBS exhibited greater fluctuation amplitude, which is consistent with the standard deviation performance characteristics analyzed previously, while the strain demodulation results of DTA-RBS maintained better stability and consistency. These comparison results indicate that DTA-RBS possesses stronger demodulation reliability and superior strain measurement performance under large strain conditions.

**Figure 10:**
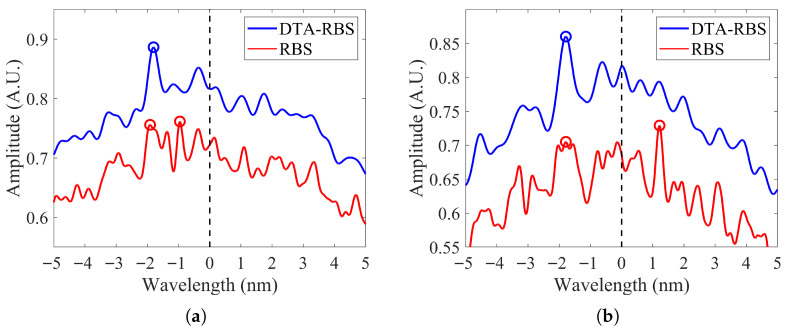
(**a**) Cross-correlation results of RBS and DTA-RBS at 47.51 m. (**b**) Cross-correlation results of RBS and DTA-RBS at 47.77 m.The blue circle marks the maximum peak of the cross-correlation function of DTA-RBS. The red circle marks the maximum peak and the second largest peak of the cross-correlation function of RBS.

To thoroughly analyze the intrinsic mechanism by which DTA-RBS possesses higher strain demodulation performance compared to RBS, this study selected two anomalous points at 47.51 m and 47.77 m where RBS demodulation failed for detailed investigation. Figure 10a,b shows the cross-correlation function comparison results at these two positions respectively. In the strain region, cross-correlation results will produce wavelength shift. Spectral deformation and signal-to-noise ratio degradation caused by strain also lead to a decrease in cross-correlation peak intensity.

Figure 10a shows the cross-correlation function characteristics at the 47.51 m position. DTA-RBS successfully achieved correct strain demodulation, with its cross-correlation peak intensity reaching 0.8865, and the peak position appearing near a wavelength shift of 1800 pm, which corresponds to the applied strain level. However, the cross-correlation function of RBS exhibits distinctly different characteristics, with its maximum peak intensity of 0.7615 appearing at the incorrect position of 1300 pm wavelength shift. Near the correct wavelength shift of 1800 pm, RBS does have a peak with an intensity of 0.7559, but this peak intensity is slightly lower than the incorrect peak at 1300 pm. Since cross-correlation algorithms typically select the maximum peak as the criterion for wavelength shift determination, the correct peak information is buried in spurious peaks of sidelobes, ultimately leading to demodulation failure.

Figure 10b further verifies the same phenomenon. At the 47.77 m position, the cross-correlation peak intensity of DTA-RBS is 0.86, with the peak position correctly appearing near a wavelength shift of 1800 pm. In contrast, the maximum peak intensity of RBS is 0.7291, while near the correct wavelength shift position of 1800 pm, its peak intensity is only 0.7051, which is significantly lower than spurious peaks at other positions. In this case, the cross-correlation algorithm completely fails to correctly identify the true peak position, leading to complete demodulation failure.

Through envelope extraction and feature enhancement mechanisms, DTA-RBS effectively suppresses high-frequency noise and spurious oscillations introduced during the reconstruction process, maintaining higher correlation between the reference data and test data. This higher correlation directly manifests as greater cross-correlation peak intensity. Even under unfavorable conditions where strain causes spectral deformation and signal-to-noise ratio degradation, DTA-RBS can still maintain sufficiently strong true peak signals. Furthermore, the higher cross-correlation peak intensity of DTA-RBS means a higher signal-to-noise ratio, with the true peak having a more significant advantage over background noise and sidelobe peaks, thereby ensuring the accuracy and reliability of peak identification. In contrast, RBS, due to containing higher density peak structures and substantial high-frequency noise information, is more prone to generate strong cross-correlation responses at incorrect positions during the cross-correlation calculation process. When strain causes a decrease in spectral correlation, the true peak intensity of RBS may not be sufficiently higher than these spurious peaks, causing the correct peak information to be masked by spurious peaks of sidelobes, ultimately resulting in demodulation failure. Therefore, by enhancing cross-correlation peak intensity and reducing spurious peak interference, DTA-RBS significantly strengthens the system’s noise resistance and demodulation robustness under large strain conditions, providing strong assurance for achieving reliable distributed strain sensing.

## 4. Discussion

In this study, experiments verified that DTA-RBS can enhance the peak intensity of cross-correlation, improve the STD of distributed demodulation, and demonstrate superior demodulation performance and robustness under large strain. We attribute these performance improvements to the envelope property of DTA-RBS, which strengthens spectral feature information and intensity while suppressing high-frequency noise and anomalous oscillations. However, DTA-RBS also has potential limitations. While the envelope property of DTA-RBS suppresses high-frequency noise, it inevitably incorporates nearby peak information in the spectrum into the stronger envelope peak, which may lead to loss of spectral details. When facing large strain demodulation, the wavelength shift is significant and spectral correlation decreases, making DTA-RBS advantageous in such scenarios by providing higher robustness for large strain demodulation. When dealing with small strain, the wavelength shift is minimal, and both RBS and DTA-RBS exhibit high correlation. In such cases, more detailed information is required to distinguish spectral changes caused by small strain. The envelope property of DTA-RBS may potentially obscure fine details within the overall envelope, which could compromise detection of small strain. We believe that DTA-RBS is more suitable for applications under large strain scenarios, where it increases demodulation robustness and improves STD performance. For small strain detection, DTA-RBS still has potential limitations. We will continue to investigate in future work, seeking to balance the relationship between the envelope enhancement and spectral detail preservation of DTA-RBS, with the goal of enabling DTA-RBS to maintain good demodulation performance under large strain while preserving its detection capability for small strain.

On the other hand, DTA-RBS demonstrates greater potential in terms of algorithm complexity. DTA-RBS does not increase algorithm complexity and requires no interpolation or smoothing of the spectrum. These advantages are particularly well-suited for application in FPGA-based OFDR demodulation system development, enabling optimization of algorithm complexity for FPGA and providing technical support for improving demodulation speed. In future work, we will implement the DTA-RBS algorithm in FPGA and conduct comprehensive research and analysis.

## 5. Conclusions

This study proposes a novel OFDR distributed demodulation optimization algorithm based on discrete-time analytic signals applied to Rayleigh backscattering spectrum reconstruction. The method establishes the mathematical framework of discrete-time analytic signals and develops a practical implementation approach that leverages the frequency-domain characteristics of analytic signals without requiring conjugate symmetric data, thereby reducing addressing ranges by half in FPGA-based implementations. The fundamental mechanism underlying the proposed approach is the envelope property of analytic signals, which effectively suppresses high-frequency noise, spurious oscillations, and reconstruction artifacts introduced during sliding window truncation, zero-padding, and inverse fast Fourier transform operations. Through systematic experimental validation across multiple dimensions, the DTA-RBS method demonstrates superior performance compared to conventional RBS reconstruction. Cross-correlation intensity analysis reveals that DTA-RBS achieves an average peak intensity of 0.9527, compared to 0.9096 for RBS, representing a consistent enhancement across the entire fiber length. Standard deviation measurements in unstrained fiber sections demonstrate a 63% improvement, with DTA-RBS achieving 2.8267 pm compared to 7.7808 pm for RBS, maintaining enhanced demodulation stability even under degraded signal-to-noise ratio conditions. Furthermore, distributed strain demodulation experiments under large strain loading conditions demonstrate that DTA-RBS exhibits more stable and reliable strain demodulation capability. DTA-RBS possesses cross-correlation peaks with higher signal-to-noise ratios, while RBS peaks are more susceptible to being obscured by sidelobe noise and spurious peaks. DTA-RBS demonstrates superior robustness and noise resistance. These improvements collectively establish that the discrete-time analytic signal approach provides a theoretically rigorous and practically effective optimization pathway for enhancing OFDR sensing accuracy, stability, and robustness, offering substantial advantages for high-precision distributed optical fiber sensing applications.

## Data Availability

The raw data supporting the conclusions of this article will be made available by the authors on request.
